# Ectopic nasal tooth: A case report

**DOI:** 10.1016/j.ijscr.2021.106459

**Published:** 2021-09-28

**Authors:** Nawaf Alfayez, Salwa AlRashed ALHumaid, Abdulrhman Alfayez

**Affiliations:** King Abdullah International Medical Research Center/King Saud bin Abdulaziz University for Health Sciences, ENT Division, Department of Surgery – King Abdulaziz Medical City- NGHA, Saudi Arabia

**Keywords:** Nasal tooth, Supernumerary teeth, Paranasal CT, Foreign body, Nasal obstruction, Case report

## Abstract

**Introduction and importance:**

The ectopic eruption of the teeth into the nasal cavity is a rare phenomenon. It is mostly found incidentally or with nasal symptoms.

**Case presentation:**

A 32-year-old male patient presented with nasal obstruction and recurrent epistaxis. Nasal endoscopy revealed a mass in left nasal floor along with septal deviation and right inferior turbinate hypertrophy. Sinus CT confirmed the same clinical finding with emphasis on the mass being a foreign body mostly consistent with a tooth. Septoplasty, inferior turbinoplasty, and endoscopic removal of the nasal tooth were performed. The patient tolerated the procedure well with improvement in nasal symptoms.

**Clinical discussion:**

The exact etiology of supernumerary teeth is still unclear. There are different clinical presentations that can occur; however, the intranasal tooth can be asymptomatic or cause a variety of signs and symptoms. The diagnosis of nasal teeth is usually made by the clinical and radiographic findings, and removal of the nasal teeth is generally advised to alleviate the symptoms and prevent complications.

**Conclusion:**

Ectopic eruption of the teeth into the nasal cavity is a rare form of supernumerary teeth. Thus, crucial attention to the clinical, radiological and histopathological examination should be taken for more accurate diagnosis and thus appropriate management in case of nasal obstruction or recurrent epistaxis.

## Highlights

• Ectopic eruption of the teeth into the nasal cavity is a rare form of supernumerary teeth.
• It is most likely to be found incidently.
• It is very important to be aware of the clinical and imaging features of nasal teeth for better diagnosis and treatment

## 1 Introduction

The ectopic eruption of the teeth into the nasal cavity is a rare form of supernumerary teeth. The etiology of nasal teeth is unclear. Theories explaining it include developmental disturbances, inflammatory reactions, genetic factors, and trauma [1]. The clinical presentations can be different; however, the intranasal tooth can be asymptomatic or cause a variety of signs and symptoms, including facial pain, nasal obstruction, headache, epistaxis, rhinorrhea, and external nasal deformities [2]. It is usually found during routine clinical and radiographic examinations. We report the occurrence of this condition of an ectopic tooth found in nasal cavity in Riyadh, Saudi Arabia.

This project has been reported according to the SCARE criteria [3].

## 2 Case presentation

**Fig. 1:**
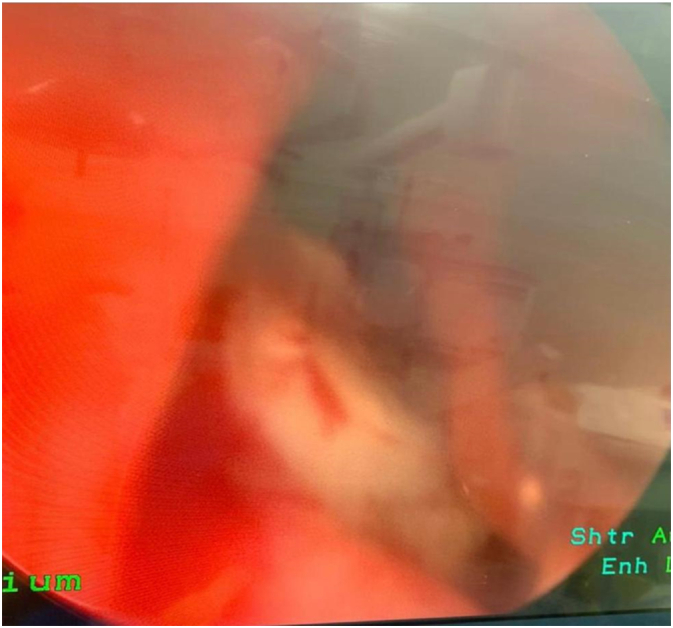
Endoscopic examination of the nose showing the nasal tooth.

**Fig. 2:**
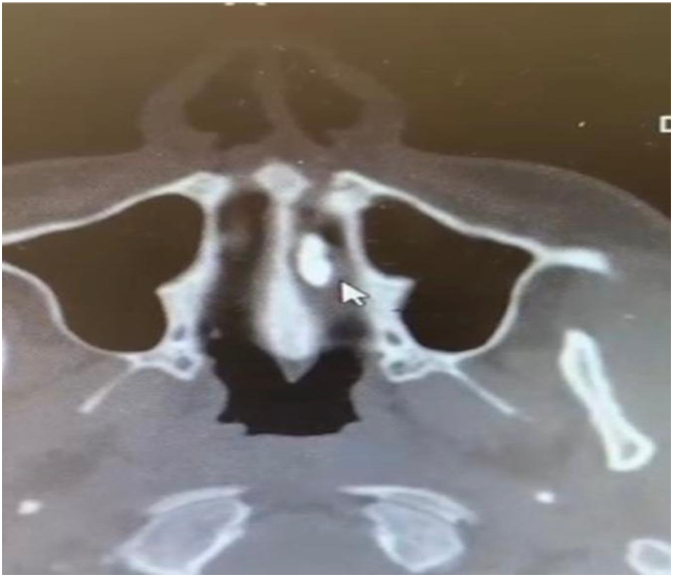
CT scan axial cut bone window showing the nasal tooth.

**Fig. 3:**
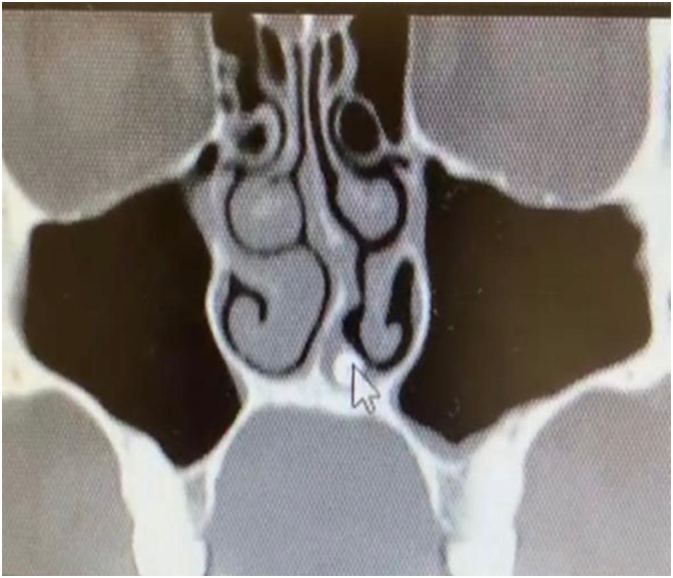
CT scan coronal cut showing the nasal tooth.

**Fig. 4:**
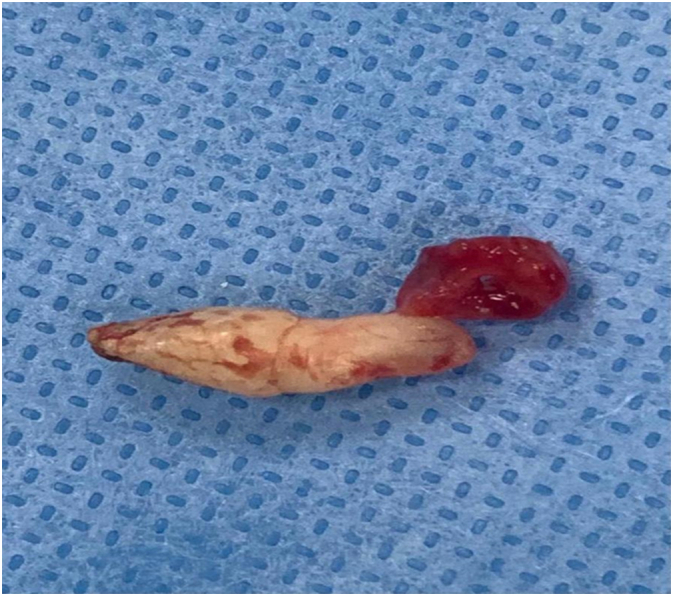
Photograph of the surgically removed ectopic tooth.

A 32-year-old male attended to the otorhinolaryngology clinic with a presentation of nasal obstruction, mouth breathing, and recurrent nasal bleeding. Regarding the history of the patient, he was medically free with no previous surgeries. He denied any smell disturbances, postnasal drip or nasal trauma. On clinical endoscopic nasal examination there was marked septal deviation to the left side with a spur and a whitish transparent mass seen on the floor of the left nasal cavity covered with mucosa (Fig. 1). CT of the paranasal sinus was done for the patient, and it showed a foreign body with a size of 1.4 × 0.4 × 0.4 cm seen within the left inferior nasal meatus likely part of a tooth, the nasal septum was deviated to the left side, hypertrophied right nasal turbinate, and the paranasal sinuses were clear (Fig. 2, Fig. 3). The patient went for septoplasty, inferior turbinoplasty, and endoscopic removal of the nasal tooth (Fig. 4), which was performed by an otorhinolaryngology surgeon with no assistance from oro-maxillofacial surgery team. He tolerated the procedure well with no post-operative complications. No histopathological confirmation of the foreign body was needed since it appearance was consistent with that of a teeth. During his follow-up few months later, patient's nasal symptoms improved markedly. Examination revealed patent nasal cavities bilaterally with no adhesions. With his clinical improvement, no repeated imaging was required. Thus decreasing his radiation exposure and financial cost-effectiveness.

## 3 Discussion

The incidence of supernumerary teeth generally affects 0.1–1% of the population [4]. The most common location is the upper incisor area, known as the mesiodens. The etiology of supernumerary teeth is unclear. Theories explaining it include developmental disturbances, inflammatory reactions, genetic factors, and trauma. The clinical presentations can be different; however, the intranasal tooth can be asymptomatic or cause a variety of signs and symptoms, including facial pain, nasal obstruction, headache, epistaxis, rhinorrhea, and external nasal deformities. Complications of nasal teeth include rhinitis cases with septal perforation, aspergillosis, and naso-oral fistula [5].

The diagnosis of nasal teeth is made by the clinical and radiologic findings. Clinically, an intranasal tooth is seen as a white mass in the nasal cavity surrounded by granulation tissue and debris [1]. Radiologically, the nasal teeth appear as radiopaque lesion with the same attenuation as that of the oral teeth. With the bone window setting, the central radiolucency, which is correlated with the pulp cavity, may have a spot or slit, depending on the orientation of the teeth [6]. The soft tissue surrounding the radiopaque lesion is consistent with granulation tissue found on clinical and pathologic examinations.

The differential diagnosis of nasal teeth includes radiopaque foreign body, rhinolith, inflammatory lesions due to tuberculosis, syphilis or fungal infection with calcification, benign tumors, including osteoma, calcified polyps, and malignant tumors, such as chondrosarcoma and osteosarcoma [7]. However, the computed tomography (CT) findings of tooth-equivalent attenuation and a centrally located cavity are highly discriminating features that help to confirm the diagnosis.

Removal of nasal teeth is generally advised to alleviate the symptoms and prevent complications. When an extra tooth is in the nasal cavity, the procedure is usually a minor operation. When a tooth has a bony socket in the floor of the nose, it may be extremely difficult to extract [8]. CT scan is useful to evaluate the depth of the eruption site.

Follow-up with repeated nasal endoscopy and paranasal sinus CT may confirm the proper healing process and resolution of the symptoms.

## 4 Conclusion

Nasal teeth occur from the ectopic eruption of supernumerary teeth. They are usually misdiagnosed because such teeth are surrounded by hypertrophic nasal mucosa or granulation tissue, however diagnosing it is not difficult, CT scanning is helpful in planning their management.

This is the only case reported in Saudi Arabia. Therefore, it is very important to be aware of the clinical and imaging features of nasal teeth.

## Sources of funding

None.

## Ethical approval

Research has been approved by the research ethical committee of King Abdullah International Medical Research Center.

## Consent

Written informed consent was obtained from the patient for publication of this case report and accompanying images. A copy of the written consent is available for review by the Editor-in-Chief of this journal on request.

## Author contribution

Nawaf Alfayez: Data collection, data analysis & interpretation and writing the paper.

Abdulrhman Alfayez: Study concept & design, data collection, data analysis & interpretation and writing the paper.

Salwa AlRashed AlHumaid: Data analysis & interpretation and writing the paper.

## Research registration

Not applicable.

## Guarantor

Abdulrhman Alfayez.

## Provenance and peer review

Not commissioned, externally peer-reviewed.

## Declaration of competing interest

The authors declare that they have no competing interests.
